# Potential of carvacrol as plant growth-promotor and green fungicide against fusarium wilt disease of perennial ryegrass

**DOI:** 10.3389/fpls.2023.973207

**Published:** 2023-02-10

**Authors:** Hamza Saghrouchni, Azeddin El Barnossi, Ibrahim Mssillou, Isilay Lavkor, Tahsin Ay, Mohammed Kara, Abdullah A. Alarfaj, Abdurahman Hajinur Hirad, Hiba-Allah Nafidi, Mohammed Bourhia, Isil Var

**Affiliations:** ^1^ Department of Biotechnology, Institute of Natural and Applied Sciences, Çukurova University, Adana, Türkiye; ^2^ Laboratory of Biotechnology, Environment, Agri-Food and Health, Faculty of Sciences Dhar El Mehraz, Sidi Mohammed Ben Abdellah University, Fez, Morocco; ^3^ Laboratory of Natural Substances, Pharmacology, Environment, Modeling, Health & Quality of Life, Faculty of Sciences Dhar El Mehraz, Sidi Mohamed Ben Abdallah University, Fez, Morocco; ^4^ Mycology unit Biological Control Research Institute, Adana, Türkiye; ^5^ Laboratory of Biotechnology, Conservation and Valorisation of Natural Resources, Faculty of Sciences Dhar El Mehraz, Sidi Mohamed Ben Abdallah University, Fez, Morocco; ^6^ Department of Botany and Microbiology College of Science, King Saud University, Riyadh, Saudi Arabia; ^7^ Department of Food Science, Faculty of Agricultural and Food Sciences, Laval University, Quebec City, QC, Canada; ^8^ Laboratory of Chemisty and Biochemistry, Faculty of Medicine and Pharmacy, Ibn Zohr University, Laayoune, Morocco; ^9^ Department of Food Engineering, Faculty of Agriculture, Çukurova University, Adana, Türkiye

**Keywords:** *Lolium perenne*, perennial ryegrass, fusarium wilt, carvacrol, bio-fungicide, plant growth promotor

## Abstract

Perennial ryegrass (*Lolium perenne* L.) is a valuable forage and soil stabilisation crop. Perennial crops have long been associated with good environmental performance and ecosystem stability. Vascular wilt diseases caused by *Fusarium* species are the most damaging plant diseases affecting both woody perennials and annual crops. Therefore, the aim of the present study was the assessment of the preventive and growth-promoting effects of carvacrol against *Fusarium oxysporum*, *F. solani*, and *F. nivale* (phylogenetically analyzed on the basis of internal transcribed spacer (ITS) regions) causing vascular wilt of ryegrass *in vitro* and under greenhouse conditions. To accomplish this aim, various parameters were monitored including coleoptile development, rhizogenesis, the incidence of coleoptile lesions, disease index, the visual appearance of ryegrass health, ryegrass organic matter and soil fungal load. The results obtained showed that *F. nivale* was highly harmful to ryegrass seedlings compared to other *Fusarium* species. Furthermore, carvacrol with 0.1 and 0.2 mg/mL protected significantly the seedlings against Fusarium wilt diseases both *in vitro* and in the greenhouse. Simultaneously, carvacrol also functioned as a seedling growth promoter, as is reflected in all monitored parameters, such as the recovery of seedling height and root length, and the development of new leaf buds and secondary roots. Carvacrol proved to be effective plant growth promoter and a bio-fungicide against Fusarium vascular diseases.

## Introduction

Perennial ryegrass (*Lolium perenne* L.) is a cool-season grass grown throughout temperate regions of the world (Wilkins & Humphreys, 2003; Wiering et al., 2021). Perennial plants have long been associated with good environmental performance and improved ecosystem health, perennials protect and stabilizer the soil against wind and water erosion, while increasing soil quality and organic matter thanks to biomass accumulation (Blanco-canqui, 2010). An increased proportion of perennials increase biodiversity by providing habitat for animals and insects. Additionally, perennial crops can increase the quantity and diversity of mineral nutrients available in the rhizosphere by establishing complex and often long-term relationships with the microbiome (Davis et al., 2010; Nehls et al., 2010). Perennial grasses with their large and active root system, play a crucial role in scavenging available nutrients and preventing them from leaching away where they may become pollutants (Heaton et al., 2010). Further, though a popular species in the turf industry, perennial ryegrass has desirable grazing characteristics and high nutritive value (Sampoux et al., 2011). Therefore, the main role of this species is to provide forage for ruminant animals. Consequently, perennial ryegrass forage breeders have focused on improving biomass, nutritive value, persistence, disease resistance, and winter-hardiness (Sampoux et al., 2011).

Vascular wilt diseases caused by fungal pathogens are the most damaging plant diseases that affect both woody perennials and annual crops (Boutaj et al., 2022). The *Fusarium* genus is one of the utmost complex and adaptive genus in the Eumycota (Gordon, 2017). Fusarium species which cause destructive vascular wilts, rots, and damping-off diseases, are ubiquitous soil-borne pathogens of a wide range of horticultural and food crops (Bodah, 2017; Saghrouchni et al., 2021). In addition to the damages caused in the pre-harvest period, many *Fusarium* species are capable of producing mycotoxins in agricultural and food commodities, as well as in perennial forage (Juraschek et al., 2022). These phytopathogens attack the vascular system made up of xylem arteries, tracheary elements that transport water and minerals from the roots to the photosynthetic organs, and phloem elements, which transport organic photosynthesis products (Agrios, 2005). High number of harmful mycotoxins produced by Fusarium can be commonly detected in ruminant diets, such as deoxynivalenol and fumonisins, which can interfere with ruminant animals and human health (Escrivá et al., 2015; Gallo et al., 2021).

*Fusarium* spp., *Microdochium* spp., and *Neocosmospora* spp. can cause destructive vascular wilt disease. *Fusarium nivale* (Fr.) Samuels and I.C. Hallet, *Fusarium oxysporum* described by Schltdl., and *Fusarium solani* (Mart.) L. Lombard and Crous, three species owning to the “Fusarium complex” have devastating effects on species belonging to wild gramineae, *Lolium perenne* L. (ryegrass) (Saghrouchni et al., 2021). These species usually limit the production of economically important crops since these strains attack several types of plants especially gramineae family including rice, maize, wheat, barley, oats, and rye, and therefore induce significant losses especially when the environmental conditions are favourable (Servin et al., 2015).

To control Fusarium wilt, several strategies were used such as soil solarisation and fumigation with various pesticides. However, the presence of chemical residues is harmful to the public health and can affect the quality of soil and groundwater (Boutaj et al., 2022). These products can be used before planting to treat soil and/or during cultivation, e.g., chloropicrin and benomyl (Dorugade et al., 2021; Karki et al., 2022). Due to excessive use, some fungicides can no longer be effective towards resistant strains among fungal species (Chaves et al., 2022 **)**. Therefore, it is necessary to identify alternative weapons to control Fusarium wilt disease.

Pathogen control and disease management can only be achieved through an integrative approach in which biological control can play a major role. Nowadays, biological agents including botanical agents (Plant extracts, essential oils and their major compounds) are used as alternative solutions (Lecomte et al., 2016; Agour et al., 2020; Jawhari et al., 2021; Amrati et al., 2021). Carvacrol is a natural low-molecular weight product, this secondary metabolite derived from plants (*Thymus vulgaris* and *Origanum vulgare* described by Linnaeus). Carvacrol has been the subject of many investigations that place a higher priority on natural products with biological activities to control such diseases. This monoterpene has been shown to possess a wide range of biological effects, such as antibacterial, antifungal, insecticidal, antioxidant, antimutagenic, antigenotoxic, and antitumor (Aprotosoaie et al., 2019). Previous investigations demonstrated that carvacrol is one of the potent monoterpenes that can be used to control fungal species (Saghrouchni et al., 2021; Gonçalves et al., 2021).

Based on the previously discussed reasons, the main aim of the current paper was to evaluate the preventive and stimulatory effects of carvacrol on Fusarium wilt disease of ryegrass *in vitro* and under greenhouse conditions. For this purpose, an infestation of three fungal strains was carried out on ryegrass pre-treated with carvacrol, and several parameters were monitored including coleoptile development and rhizogenesis, incidence of lesions on coleoptiles, disease index, visual aspect of the health status, the weight of organic matter and the Fusarium load of the soil.

## Material and methods

### Chemicals

The agar medium used in this study was 3% malt extract agar (MEA) amended with 0.5% mycological peptone, and potato dextrose agar (PDA) (Biokar, France). The monoterpene carvacrol (99%) used in this study was obtained from Flagresso, Austria.

### Fungal strains

*Fusarium oxysporum*, *F. solani*, and *F. nivale* strains used in the present study were isolated from diseased leaves, stems, and roots of ryegrass and from soil samples before being identified. For morphological characterisation, macroscopic traits such as the colony appearance, colour, pigmentation and growth rate were observed on potato dextrose agar (PDA) according to Leslie & Summerell (2006). Morphological identification was also performed based on the morphological characteristics observed at optical microscope as described by Booth (1971) and Leslie & Summerell (2006).

### Fungal DNA extraction

The morphological identification was further confirmed by DNA sequencing. Single hyphal tip isolates were grown on cellophane overlain on PDA for 10 days. The mycelium was lyophilized and 10 mg of lyophilized mycelium, grinded with 5 mm iron beads. Then, fungal DNA was extracted using a DNeasy Plant Mini Kit (Qiagen, Hilden, Germany) according to the manufacturer’s protocol (Fallahi et al., 2019). The quality and quantity of the DNA obtained were evaluated by measuring the concentration (ng/μL) in a NanoDrop ND-1000 spectrophotometer (Thermo Fisher Scientific). The quality of the DNA yielded by each method was determined by gel electrophoresis in a 1% agarose gel at 90 V for 15-20 min, stained with Ethidium bromide

### Molecular identification

To confirm the identity of the fungus, ITS-5 (5`-GGAAGTAAAAGTCGTAACAAGG-3`-) and ITS-4 (5`-CCTCCGCTTATTGATATGC-3’) were used to amplify complete internal transcribed spacer (ITS) as described by White et al. (1990). The ITS region was amplified in a 25 μL reaction using 25 ng DNA, 1 x Dream Taq buffer (1.5 mM MgCl_2_), 0.3 μM pimer, 200 μM dNTP, 0.2 U Taq DNA Polymerase. The PCR conditions were as follows: 95°C for 1 min, 35 cycles of 95°C for 15 s, 55°C for 30 s, and 72°C for 90 s, and a final cycle at 72°C for 5 min. PCR products were electrophoresed on a 1% (w/v) agarose gel in 1x THE (20 mM Tris-HEPES, pH 8.06) at 90 V for 1 hour. The product was purified and then sequenced. The nitrogen base sequence was analyzed using an automated DNA sequencer. Sequencing data were trimmed and assembled using ChromasPro program version 1.5. The program Molecular Evolutionary Genetic Analysis software, ver. 5.0 (MEGA4.0; http://www.megasoftware.net) was performed in order to edit and align the sequence files, which were manually adjusted. In order to assess the relationships between the major taxa, ambiguous parts of the ITS regions were removed from further analysis and more conserved and alignable parts of the region and all genes were used to generate phylogenetic trees containing representative taxa from major groups. The assembled data were BLASTED with genomic data that has been registered in NCBI/National Center for Biotechnology Information (http://www.ncbi.nlm.nih.gov/BLAST/). The aligned sequences were BLAST in GenBank database, to identify all the strains. In this study, phylogenetic tree was generated using maximum parsimony (MP) in MEGA5.0 (Tamura et al., 2011). Bootstrap values for the maximum parsimony tree (MPT) were calculated for 1,000 replicates (Felsenstein, 1985). The edited ITS sequences were compared with other available Fusarium species sequences in the GenBank. Furthermore, the sequences of some known species were downloaded from GenBank and used to reconstruct a combined ITS region and phylogenetic trees.

### Preparation of fungal inoculants

Sporulation was obtained by culturing the fungal strains in a MEA medium after 7 days of incubation at 28°C (Pujol et al., 1997). Afterwards, the spores were collected by flooding the plate with 1 mL water, including 0.05% Tween 20, using a sterile spread rod. Then, the number of spores was counted manually with a haemocytometer (Merck, Germany) with light microscopy (Optika, Italy) before being diluted to an inoculum of about 10^6^ spores/mL (Noman et al., 2016).

### Plant material

Ryegrass seeds of the English Ryegrass variety were kindly offered by the technical service of the Golf Royal Dar Es-salaam, Rabat, Morocco.

### Culturing substrate

The substrate used for the cultivation of the ryegrass consists of 100% vegetable soil. Topsoil sieved through a 2-mm sieve was autoclaved twice at 121°C/0.1MPa for 30 minutes.

### Evaluation *in vitro* of the growth-promoting and preventive effects of carvacrol on ryegrass

Thirty seeds were disinfected with 0.5% sodium hypochlorite solution for 20 min and then rinsed twice with sterile distilled water for 10 min and dried before being transferred to Petri plates containing sterile layers of filter paper (Kordali et al., 2007; Kordali et al., 2008). Afterward, each group received 1 mL of sterile water daily. After seeds germination, 1 mL of 99% carvacrol diluted in agar 0.2% (1:10) was dispensed into the plates. Afterwards, the infestation of the groups was carried out by adding 1 mL of spores’ suspension (10^6^ spores/mL) of the *F. oxysporum*, *F. solani*, and *F. nivale* and with a consortium (1:1:1) of all the spores’ strains. The experiment was carried out for 4 weeks under ambient temperature (28 ± 2°C). The experimental protocol has been presented in the **Table 1**. Plant height (PH), root length (RL), percentage of second leaf appearance (SL) and percentage of second root appearance (SR) were measured weekly. Moreover, the lesions developed on the coleoptile were also noted and the incidence of the disease (I) was estimated by the following equation (Mironenko, 1960):

**Table 1:** Experimental protocol for the carvacrol treatment *in vitro*.

Groups distribution	
**Negative control**	30 uninfested and untreated seeds
**Positive control**	30 infested and untreated seeds
**Group I**	30 infested seeds + 1 mL of 0.05 mg/mL carvacrol
**Group II**	30 infested seeds + 1 mL of 0.10 mg/mL carvacrol
**Group III**	30 infested seeds + 1 mL of 0.20 mg/mL carvacrol


I(%)=(n1−n2)/n1  x  100

Where n1 and n2, respectively, represent the total number of seeds per Petri plate and the number of seeds with unaltered coleoptile of seedlings.

### Evaluation of the preventive effect of carvacrol on ryegrass grown in soil under greenhouse conditions

One gram of ryegrass’ seeds (approximately 200 seeds) was seeded into pots containing 150 g of topsoil. Each group received a constant volume of 15 mL (in order to wet the soil completely) of carvacrol (diluted in agar 0.2% (1:10)) twice a week. After that, infection was carried out with 15 mL of the spores’ suspension (10^6^ spores/mL) of *F. oxysporum*, *F. solani*, and *F. nivale* and with a mixture of all strain’s spores. The experimental protocol has been presented in the **Table 2**. The evolution of the fungal charge of Fusarium was followed once a week using the serial dilution technique. One gram from each sample was transferred to test tube containing 9 mL of 0.9% NaCl. From this solution, a series of dilutions (10^-2^ to 10^-6^) were made (El Barnossi et al., 2019). From each dilution 0.1 mL was spread on Petri plates containing MEA medium. The plates were incubated at 27°C for 4 days. Colonies of Fusarium species were then counted by the enumeration technique. Consequently, plates containing 15 to 150 colonies were selected for counting and the result was expressed in log (CFU/g) of three replicates (Ribeiro et al., 2020). As regards the virulence of each fungal strain, it was estimated after 7 weeks based on the disease index (DI) technique using the 0-4 scale established by Hernandez et al. (1998) which was based on visible symptoms on each seedling, where code 4: dead turf, code 3: 67 to 99% affected plant, code 2: 34 to 66% affected plant, code 1: 1 to 33% affected plant and code 0: no visible symptoms. The experiment was carried out under greenhouse conditions using both normal topsoil and disinfected topsoil.

**Table 2:** Distribution groups for the carvacrol treatment under greenhouse.

Distribution groups	
**Negative control**	200 uninfested and untreated seeds
**Positive control**	200 infested and untreated seeds
**Group I**	200 infested seeds + 15 mL of 0.05 mg/mL carvacrol
**Group II**	200 infested seeds + 15 mL of 0.10 mg/mL carvacrol
**Group III**	200 infested seeds + 15 mL of 0.20 mg/mL carvacrol

### Measurements of fresh and dry weights

To determine the fresh shoots and roots weight, the shoots and roots were carefully washed under running tap water. Prior to weight determination, excess moisture was blotted using paper towels, and the weight was determined. While for the dry weight determination, the samples were dried in an oven at 70°C for 72 h till constant weight was reached after the dry weight was determined (Huang et al., 2017).

### Statistical analysis

Quantitative results were expressed as means, of triplicate experiments ± SD (standard deviation). To determine statistical significance, Two way-ANOVA and Tukey’s multiple range tests at p< 0.05 were performed using GraphPad Prism 8.0.1 (Graph Pad Software Inc., San Diego, USA).

## Results

### Molecular identification

Molecular identification was confirmed by sequencing the ITS. BLAST analysis using ITS sequences revealed a threshold of 100% similarity between the sequences from the isolates in this study and sequences from strains which were previously deposited in Genbank. The ITS sequences were deposited in GenBank as: *F. solani* (accession no. OP810954)*, F. oxysporum* (accession no. OP824842), and *F. nivale* (accession no. OP810953).

**Figure 1:**
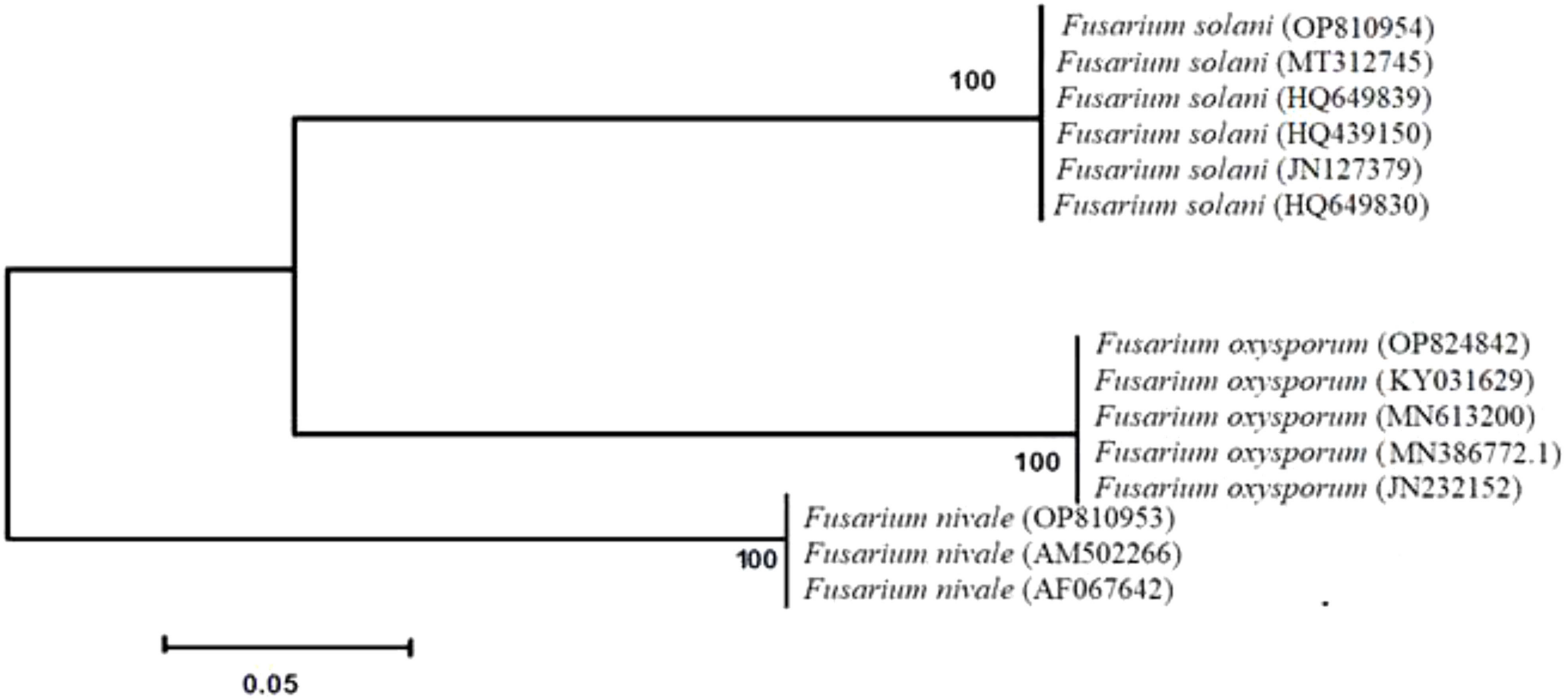
Phylogenetic analysis obtained by sequence analysis of *Fusarium* isolates 18S rDNA-ITS-region. Bootstrap tests were performed with 1,000 replications.

All strains isolated from soils and ryegrass represented two different lineages (**Figure 1**). The isolates of *F. solani* and *F. oxysporum* were divided into subgroups. *F. nivale* was included in other branches. Phylogenetic tree demonstrated that all strains are closely related with a strong bootstrap support (100%). All isolates described as *F. solani, F. oxysporum* and *F. nivale*, based on morphological characteristics, were identified as *F. solani, F. oxysporum* and *F. nivale* through molecular analyses.

### Evaluation *in vitro* of the growth-promoting and preventive effects of carvacrol on ryegrass

**Table 3** shows the results of the effect of carvacrol treatment on the coleoptile growth and on rhizogenesis of ryegrass *in-vitro*. After infestation of the seedlings with fungal spores, Fusarium significantly reduced plant height and root length for all tested strains compared to the negative control, especially *F. nivale* which was the most virulent on ryegrass seedlings. In contrast, after treatment, plant height and root length recovered well with all tested concentration of carvacrol. As a consequence, the measurement values of the treated plants were almost equal to those of the negative control and sometimes better.

**Table 3:** The effect of carvacrol treatment on the coleoptile growth and on rhizogenesis of the ryegrass *in vitro*.

F. nivale	PH (cm)	RL (cm)	SL (%)	SR (%)
**Negative control**	7.77 ± 0.25^a^	2.10 ± 0.10^b^	0.00^d^	10.00 ± 1.00^b^
**Positive control**	1.70 ± 0.20^a^	0.47 ± 0.05^a^	1.70 ± 0.20^a^	1.37 ± 0.47^a^
**0.05 mg/mL**	5.70 ± 0.10^b^	2.27 ± 0.15^b^	3.33 ± 0.58^b^	13.00 ± 10.00^c^
**0.1 mg/mL**	6.33 ± 80.00^b^	2.23 ± 0.15^b^	16.67 ± 0.58^c^	13.33 ± 0.58^c^
**0.2 mg/mL**	5.33 ± 0.05^b^	2.23 ± 0.05^b^	3.30 ± 0.58^b^	10.33 ± 0.58^b^
*F. oxysporum*				
**Negative control**	7.77 ± 0.25^c^	2.10 ± 0.10^b^	0.00^d^	10.00 ± 1.00^b^
**Positive control**	2.60 ± 0.10^a^	1.07 ± 0.05^a^	2.90 ± 0.10^a^	2.93 ± 0.11^a^
**0.05 mg/mL**	6.60 ± 0.10^b^	2.07 ± 0.05^a^	10.33 ± 0.58^c^	20.33 ± 0.58^d^
**0.1 mg/mL**	8.13 ± 0.05^c^	1.93 ± 0.05^a^	10 ± 10.00^c^	26.67 ± 0.58^c^
**0.2 mg/mL**	6.60 ± 0.10^b^	2.03 ± 0.05^a^	23.33 ± 0.58^b^	34.00 ± 10.00^e^
*F. solani*				
**Negative control**	7.77 ± 0.25^c^	2.10 ± 0.10^b^	0.00^a^	10.00 ± 1.00^b^
**Positive control**	2.10 ± 0.10^a^	0.50 ± 0.10^a^	0.00^a^	6.67 ± 0.58^a^
**0.05 mg/mL**	7 ± 0.00^c^	2.77 ± 0.05^b^	17.33 ± 1.15^d^	17.00 ± 1.00^d^
**0.1 mg/mL**	7.67 ± 0.58^c^	2.87 ± 0.05^b^	21.00 ± 1.00^c^	27.00 ± 1.00^c^
**0.2 mg/mL**	5.07 ± 0.11^b^	1.67 ± 0.58^ab^	10.33 ± 0.58^b^	23.67 ± 0.58^e^
Spores’ mixture				
**Negative control**	7.77 ± 0.25^c^	2.10 ± 0.10^b^	0.00^d^	10.00 ± 1.00^a^
**Positive control**	2.30 ± 0.10^a^	1.03 ± 0.05^a^	1.00 ± 0.00^a^	11.00 ± 1.00^a^
**0.05 mg/mL**	7.70 ± 0.27^b^	2.13 ± 0.05^a^	10.33 ± 0.58^b^	10.00 ± 1.00^a^
**0.1 mg/mL**	6.10 ± 3.47^b^	2.13 ± 0.05^a^	16.33 ± 3.78^c^	14.67 ± 0.58^b^
**0.2 mg/mL**	6.50 ± 0.50^b^	1.43 ± 0.05^a^	12.67 ± 0.58^b^	14.67 ± 0.58^b^

PH, plant height; RL, root length; SL, second leaf and SR, second root. Mean values (± SD, n = 3) followed by different letters in same row are significantly different (Tow-way ANOVA; Tukey’s test, p ≤ 0.05). Two results read from the same column and marked with the same letter do not differ significantly at a threshold α = 5%.

On the other hand, **Table 3** shows also the results of the carvacrol effect on plant development. According to the result portrayed by the table plants in both the positive and negative control did not develop a second leaf. However, in the treated groups between 10 and 23.33% of the plants have developed a second leaf during the experiment time. In addition, as regards the carvacrol effect on the appearance of the second root, 10% of the plants in the negative control developed the second root. After infestation with *F. nivale*, the fungus inhibited the development of the second root, and consequently the percentage was decreased to 1.37%. Treatment with 0.05, 0.1, and 0.2 mg/mL carvacrol significantly allowed the emergence of the second root in all plants with a percentage reaching 20.33, 26.67 and 34% respectively.

**Figure 2:**
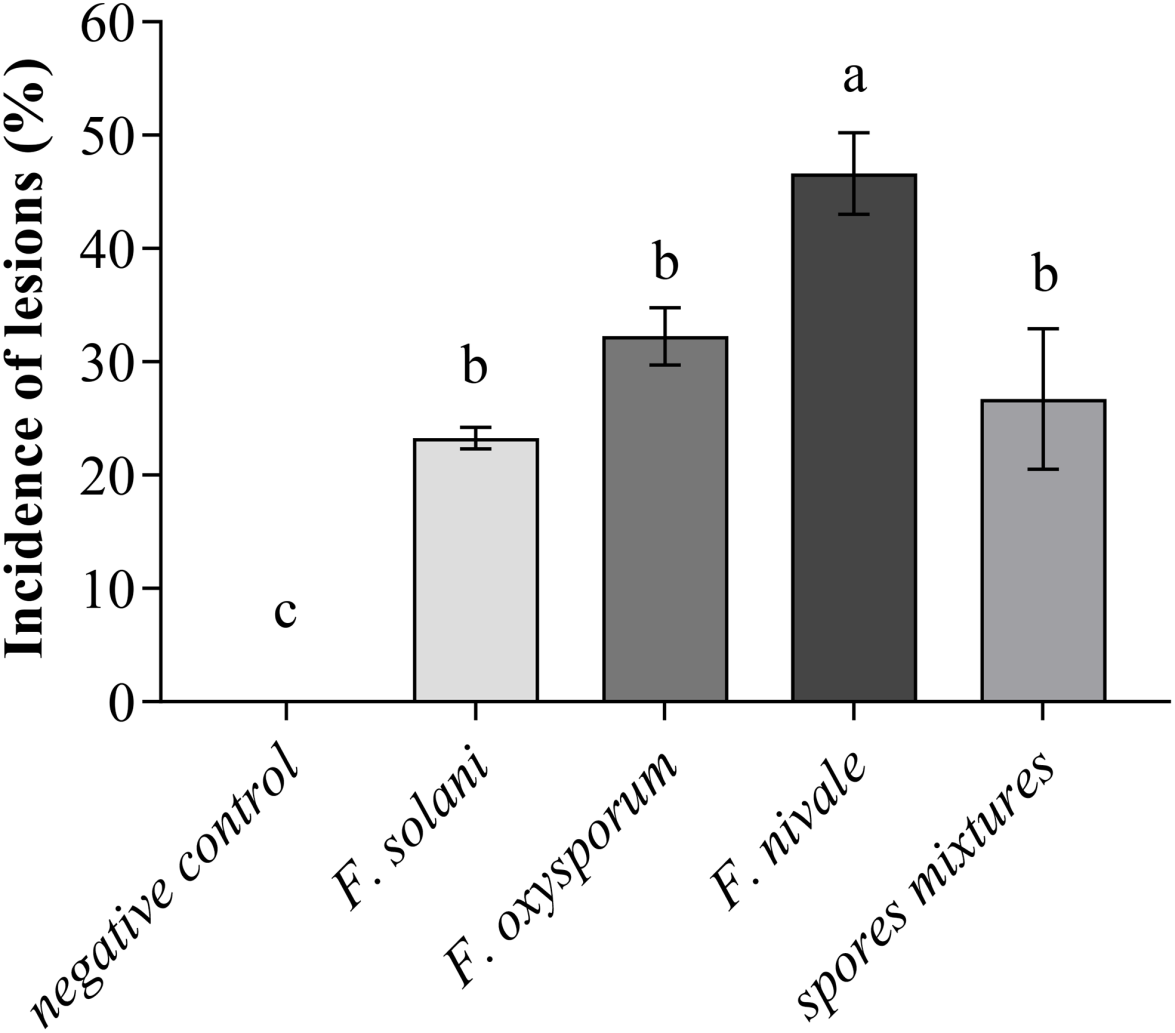
Incidence of lesions on coleoptiles of ryegrass seedlings. Means (± SD, n = 3) denoted by the same letter indicate no significant difference according to Tukey’s multiple range tests at p< 0.05.

Regarding the incidence of lesions on coleoptiles of ryegrass seedlings, the **Figure 2** shows that the incidence was high with *F. nivale* with a percentage of 50%. *Fusarium solani*, *F. oxysporum* and the spores mixtures did not induce severe lesions on coleoptiles, where the incidence percentage ranged between 23 an 33%.

According to the results, carvacrol showed protective effect on ryegrass against Fusarium wilt disease. Furthermore, carvacrol showed growth-promoting effect on both the leaves and the rhizogenesis.

### Evaluation of the preventive effect of carvacrol on ryegrass grown in soil under greenhouse conditions

**Figure 3:**
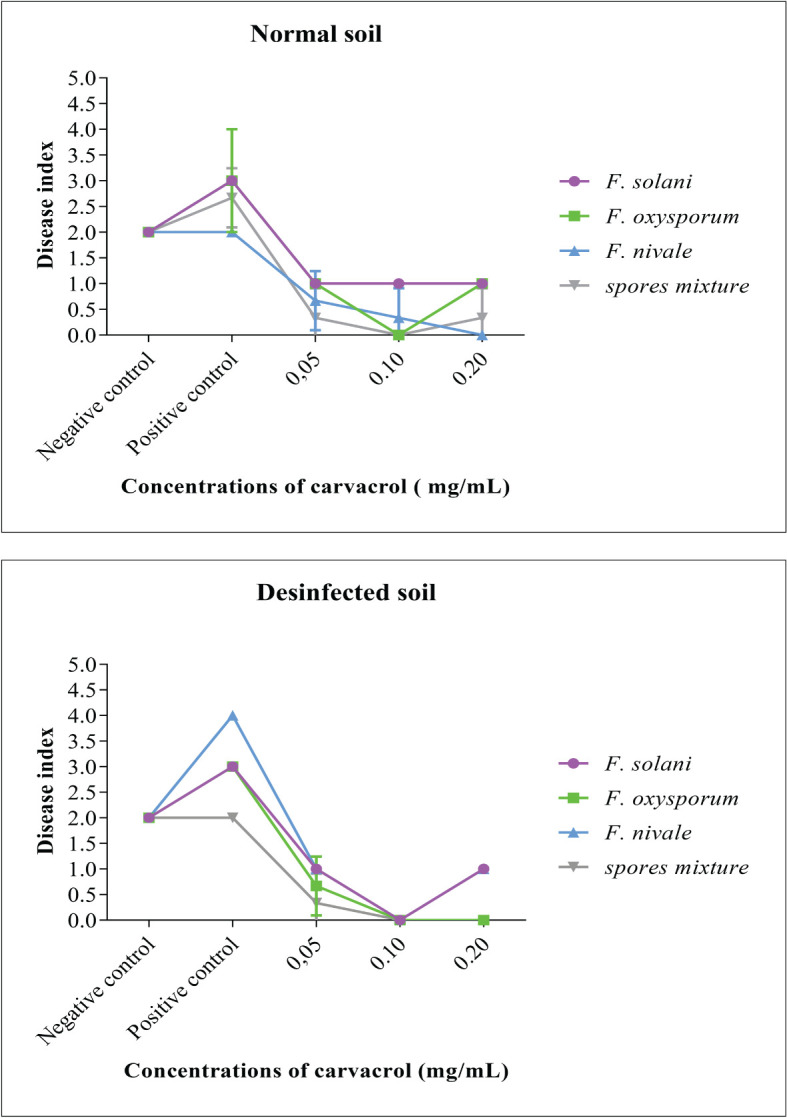
The disease index and the evolution of the average symptom severity on infested ryegrass after 7 weeks of preventive treatment with carvacrol. The results differ significantly at a threshold α = 5% (Two-way ANOVA, Turkey test). Error bars show standard deviations.

**Figure 4:**
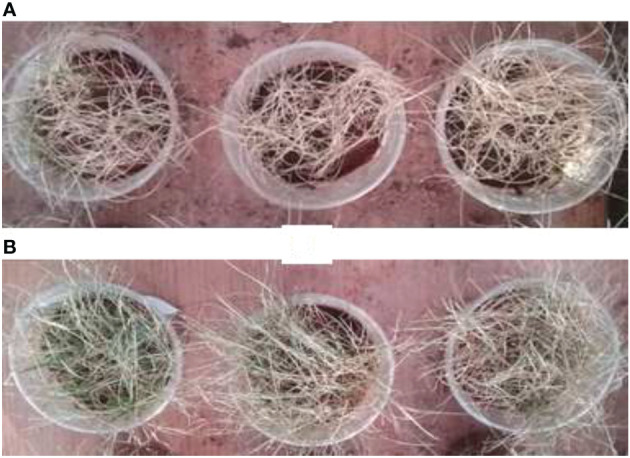
Visual appearance of the health status of ryegrass in disinfected soil. **(A)**: dead plants due to fusarium wilt disease induced by **(*F*)** *nivale* and **(B)**: negative control.

To evaluate the preventive effect of carvacrol and the virulence of the Fusarium species, the disease index (DI) was used. This index is an indication of the health status of the plant, the more it tends towards 4 the more affected the plant is. The results presented in **Figure 3** demonstrate that before infestation, the DI was equal to 2, whereas, after the infestation the DI was increased to 3 and 4. In contrast, after the treatment with carvacrol, the DI decreased to 1 and 0. In the disinfected soil, *F. nivale* - with a DI tending towards 4 - was found to be the most virulent, however in the normal soil this virulence was reduced which was reflected by the elevation of DI to 2 as the negative control. The aggresivity of *F. nivale* is manifested by the total death of almost all plants of the infested and untreated lot in the disinfected soil (**Figure 4**). Regarding the most effective concentrations of carvacrol, 0.1 and 0.2 mg/mL were shown to have a better protective effect with a DI ranging between 1 and 0 against all tested strains and in both normal and disinfected soil.

**Figure 5:**
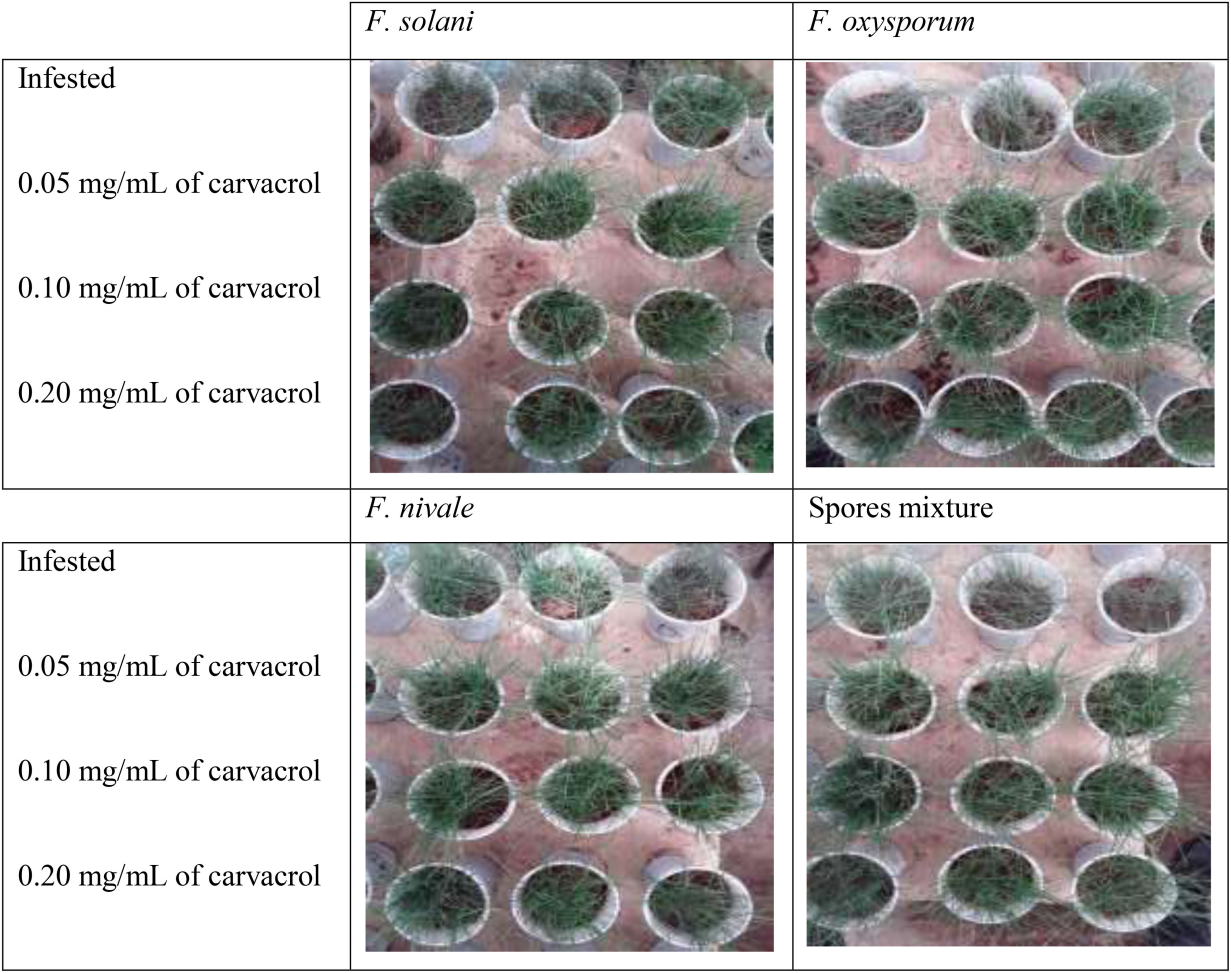
The visual appearance of the health status of ryegrass in normal soil after 4 weeks of preventive treatment with carvacrol under greenhouse conditions.

**Figure 6:**
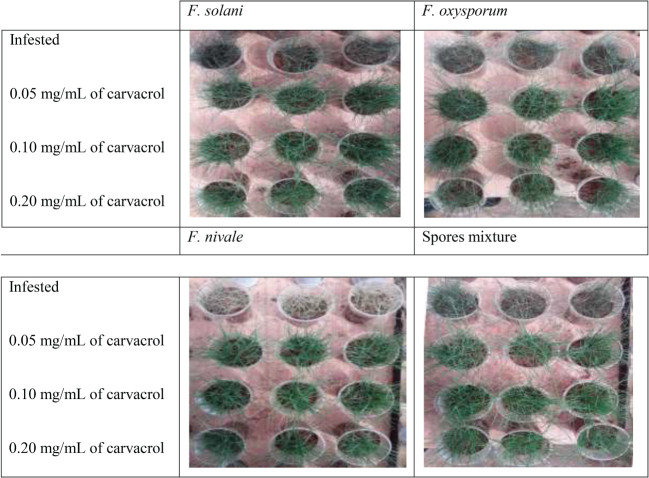
The visual appearance of the health status of ryegrass in disinfected soil after 4 weeks of preventive treatment with carvacrol under greenhouse conditions.

**Figures 5**, **6** show the appearance of the health status of all pots in the greenhouse in disinfected and normal soil. The pictures show that the plants in the infested and treated lots with carvacrol were healthier than those in the infested and untreated lots.

**Figure 7:**
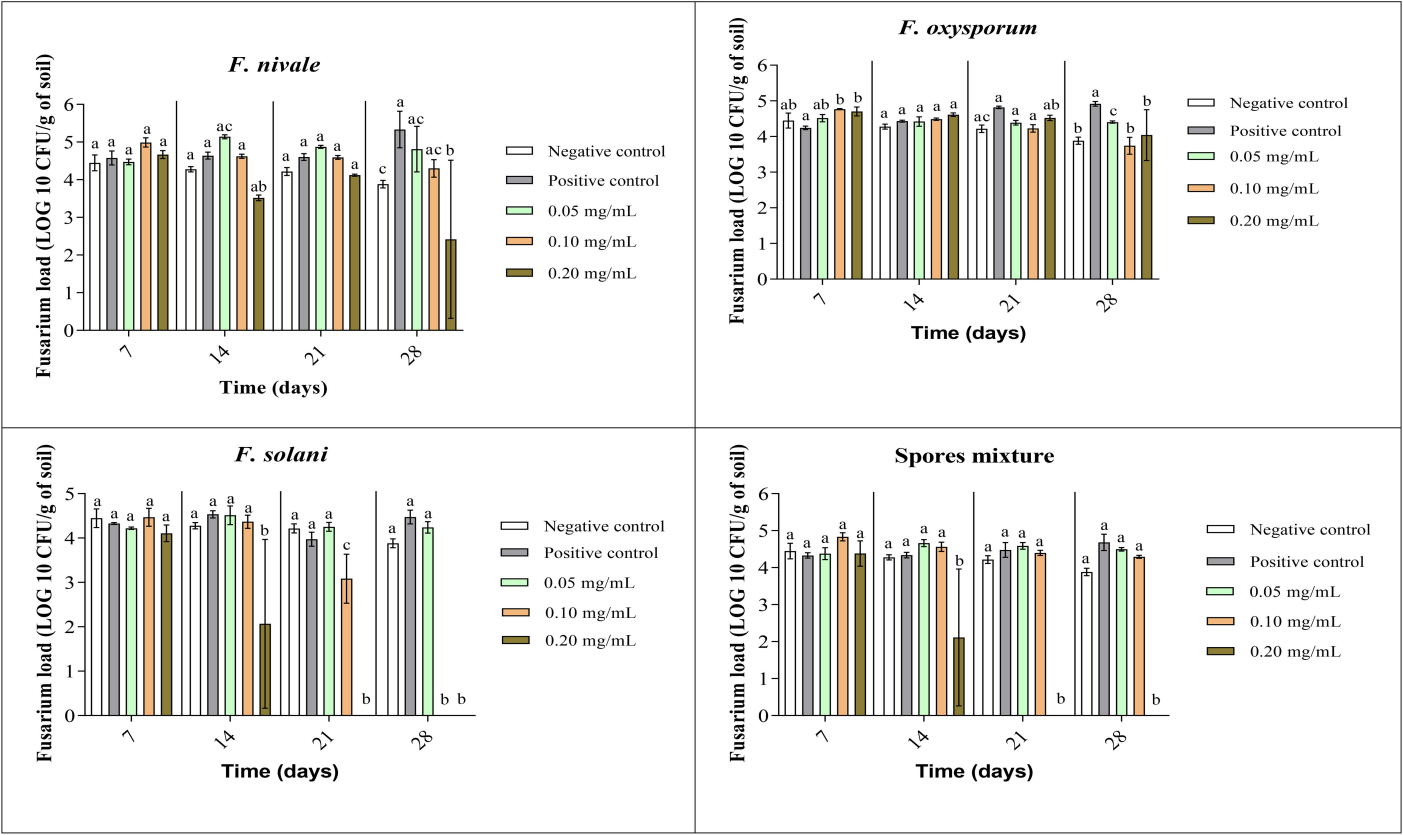
Fusarium load during treatment of ryegrass pots with carvacrol for four weeks in normal soil under greenhouse. Means (± SD, n = 3) denoted by the same letter indicate no significant difference according to Tukey’s multiple range tests at p< 0.05.

**Figure 8:**
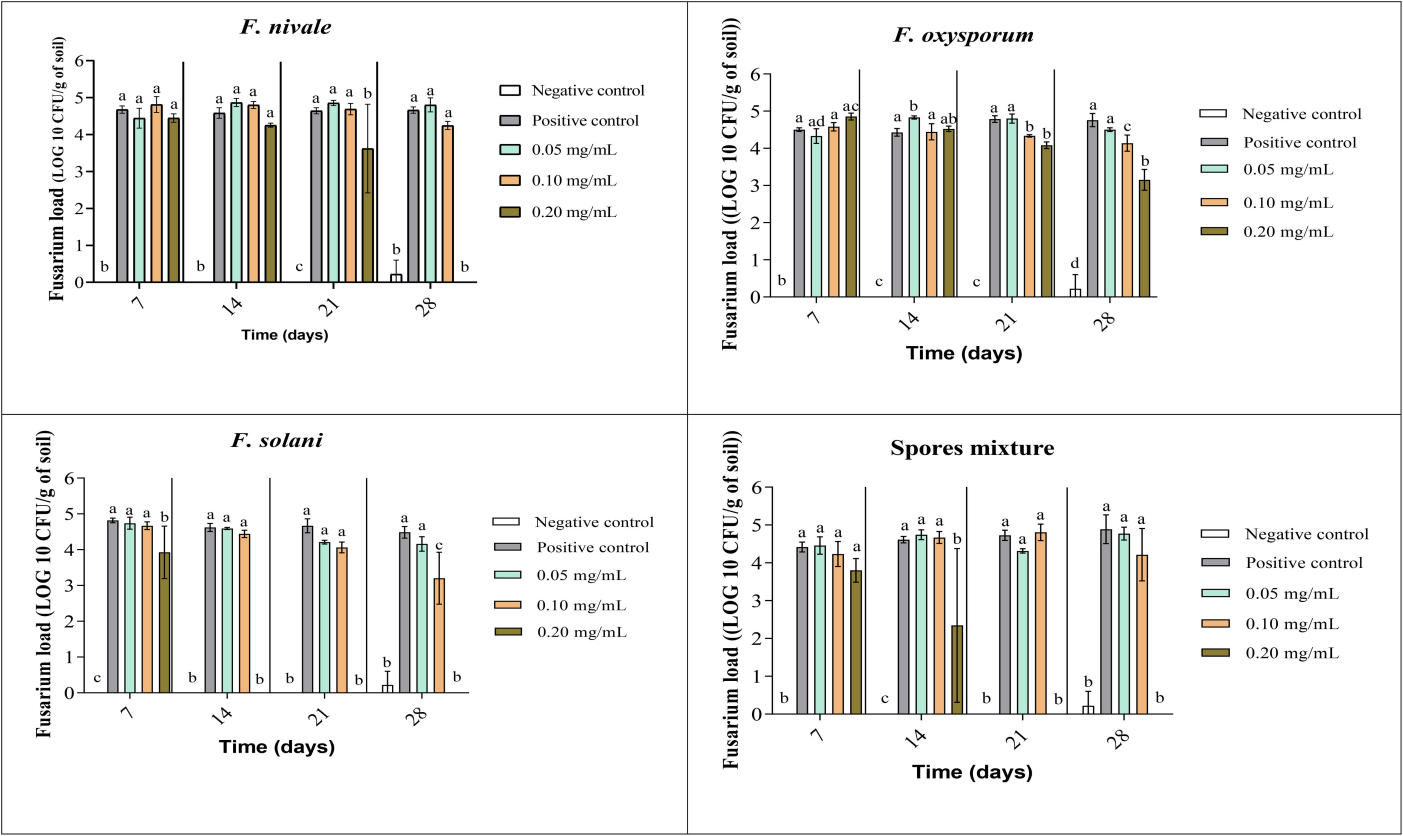
Fusarium load during treatment of ryegrass pots with carvacrol for four weeks in disinfested soil under greenhouse. Means (± SD, n = 3) denoted by the same letter indicate no significant difference according to Tukey’s multiple range tests at p< 0.05.

The fungal load of Fusarium was measured during the whole period of ryegrass cultivation. **Figures 7**, **8** show the results of the follow-up of this load in both disinfected and normal soil. According to the figures, after the infestation, the Fusarium load was much higher in the disinfected soil than in the normal soil compared with negative control. In the normal soil, after treatment, the load of *F. solani* became null from the third week of treatment with 0.2 mg/mL and at the end of the fourth week the load was nulled with 0.1 mg/mL of carvacrol. In the disinfected soil, *F. solani* was eliminated with 0.2 mg/mL of carvacrol from the second week. Furthermore, with this concentration, the fungal load of all the spores’ mixture was well eliminated after three weeks in both soil types. We noticed also that the disinfected soil remained without any apparition of fungal charge until the last week, we found a little of Fusarium charge (**Figure 8**).

### Measurements of fresh and dry weights

**Figure 9:**
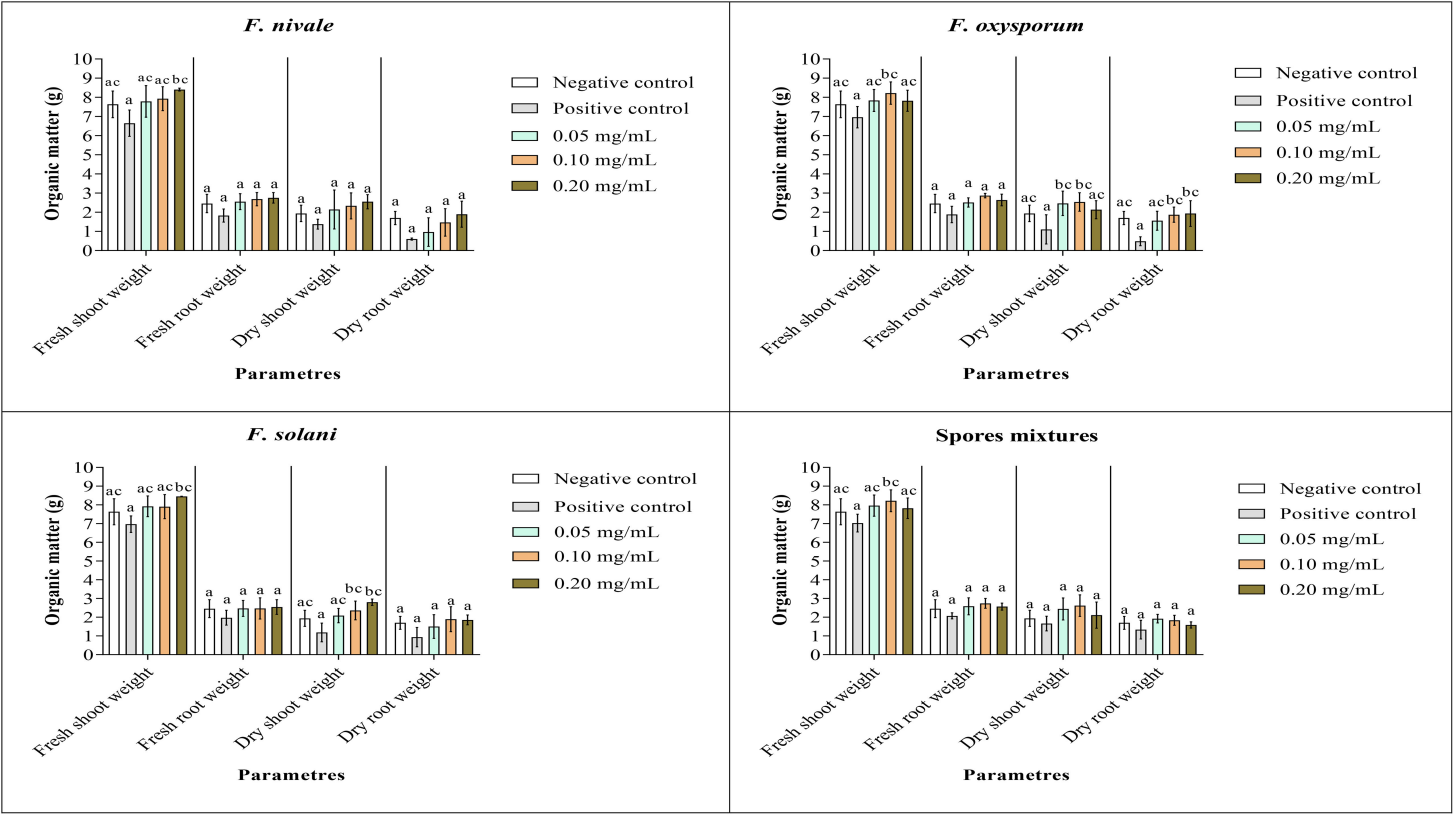
Organic matter of ryegrass after four weeks of treatment with carvacrol in normal soil under greenhouse. Means (± SD, n = 3) denoted by the same letter indicate no significant difference according to Tukey’s multiple range tests at p< 0.05.

**Figure 10:**
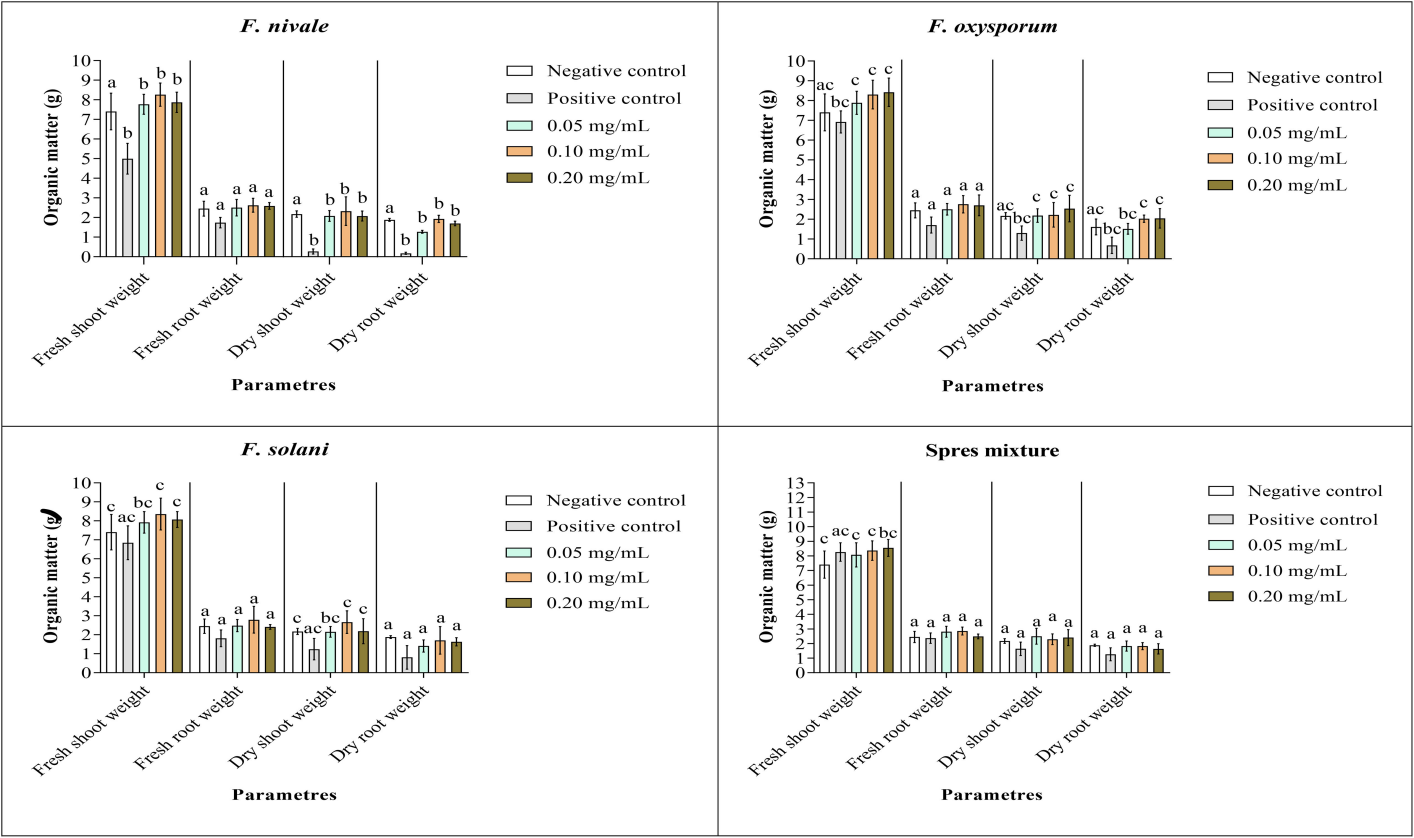
Organic matter of ryegrass after four weeks of treatment with carvacrol in disinfected soil under greenhouse. Means (± SD, n = 3) denoted by the same letter indicate no significant difference according to Tukey’s multiple range tests at p< 0.05.

**Figures 9**, **10** show the results of the quantitative evaluation of the vegetative growth variables such as fresh and dry weight of ryegrass shoots and roots after 4 weeks of treatment with carvacrol in normal and disinfected soil under greenhouse. In **Figure 9**, where we used normal soil, after infestation with Fusarium spores, the amount of organic matter has reduced significantly for all the groups and also for all growth parameters. After treatment, the amount of organic matter recovered and sometimes even increased especially with 0.1 and 0.2 mg/mL of carvacrol, compared to the negative control. On the other hand, in disinfected soil (**Figure 10**), *F. nivale* showed more damage on ryegrass. As a result, the amount of organic matter decreased from 7.4 to 4.9 g, from 2.17 to 0.26 g and from 1.87 to 0.17 g for the fresh shoots weight, dry shoots weight and dry root weight respectively.

## Discussion

From the results obtained, we found that all three strains tested negatively influenced the development and growth of coleoptiles and seedling roots. Furthermore, the incidence of lesions on coleoptiles was very high for *F. nivale*. After *in vitro* treatment with carvacrol, the height of the seedlings was restored and also the length of the roots. During the treatment we noticed that the carvacrol treated seedlings fared better than the negative control plants due to the effect of carvacrol on the development of new leaf buds and also on the emergence of secondary roots.

The results obtained were in agreement with what Boudoudou et al. (2009) reported, infestation with *F. graminearum*, *F. nivale, F. solani* and *F. semitectum* influenced coleoptile and root development of seedlings of five rice varieties. The length of coleoptiles decreased from 4.0-6.1 to 2.6-5.3 cm. The number of roots decreases from 6.0-8.4 to 4.0-7.5 and the length of roots decreases from 5.5-7.0 to 3.2-6.9 cm. This decrease was high with *F. graminearum* and *F. nivale* in contrast to *F. solani*. In the same study, the researchers evaluated the lesion incidence on coleoptiles, therefore *F. solani* did not induce lesions on coleoptiles as opposed to the other three species. The incidence was high with *F. semitectum* for two rice varieties (28.5 and 25.1%) and with *F. nivale* for one variety (29.9%). All varieties were affected by *F. graminearum* (6.5 to 21.46%). The fact that Fusarium penetrates through the roots of the plant and damages the whole plant through the vessels of the plant, is why it is called vascular disease (Shaban et al., 2018). Therefore, we thought to apply a systematic (in soil) preventive treatment in order to avoid the penetration of Fusarium into the roots and thus the establishment of the disease.

The results obtained on the disease index showed that infected plants treated with 0.1 and 0.2 mg/mL carvacrol were well protected. It should also be noted that the isolates expressed more virulence in the disinfected soil than in the normal soil. This would be in favour of a competition between the *Fusarium* and the soil flora which would exert an inhibition on Fusarium. And this was reflected by the visual appearance of the ryegrass seedlings.

The improvement of the disease index following the carvacrol treatment can be explained by the results obtained on the *Fusarium* load of the soil. Indeed, the fungal load of the soil was decreased in the function of the dose and time. These results are in accordance with other study findings, the treatment of disinfected soil with carvacrol resulted in a reduction in dose-dependence of *F. oxysporum* load. 0.8 mg/mL of carvacrol was capable of reducing the load to 0 after 3 weeks. In normal soil, the total disinfection has taken 4 weeks of treatment with the same concentration (Saghrouchni et al., 2020). In another study undertaken on soil disinfection using thymol, another major compound of *T. vulgaris* and *O. vulgare*. the treated lot with 0.5 mg/mL of thymol has shown a decrease after 21 days from 5.26 Log10 to 3.56 Log10. For the treated lot with 2 mg/mL of thymol, a total disappearance of *F. oxysporum* occurred after 14 days (Oukhouia et al., 2017).

After providing an idea of the visual and qualitative aspects of ryegrass, we assessed vegetative growth variables such as fresh and dry shoots and root weights of the ryegrass. The results of the quantitative evaluation of the vegetative growth variables showed that the amount of organic matter was recovered after being decreased after infestation and sometimes even increased compared to negative control especially with 0.1 and 0.2 mg/mL of carvacrol. These findings evidently showed that carvacrol is an effective plant growth promoter. Therefore, this monoterpene can be used as biostimulator for plant development. Nowadays, there is increasing interest in the use of naturally occurring ‘biostimulators’ for enhancing the growth of agricultural and horticultural crops (Kurepin et al., 2014). The activity of biostimulators to promote plant growth is often attributed to their ability to directly or indirectly provide mineral nutrients (mostly N, but also P, S and other macro- and micro-nutrients) to plants. Although optimal growth of plants depends on the availability of adequate mineral nutrients, that growth (and also development, including reproduction) is also regulated by plant hormones (phytohormones), including gibberellins, auxins, cytokinins, abscisic acid, ethylene and other phytohormones (Bassanezi et al., 2021; Wu et al., 2021; Liu et al., 2022). For example, auxin is involved in the stimulation of cell elongation in shoots and root initiation. Gibberellins, can also increase stem elongation. Cytokinins, which are usually found in high concentrations in meristematic regions and other areas of active growth, such as actively expanding portions of roots and stem, young leaves, developing fruits and seeds, are involved in the stimulation of cell division, shoot growth, root development, leaf expansion and the inhibition or slowing of leaf or organ senescence.

Carvacrol and other plant products can be useful in crop production due to their antimicrobial and growth-promoting potential. On the other hand, in livestock production, the use of medicinal plants is gaining more recognition as a result of numerous efforts (Oluwafemi et al., 2020). The worldwide interest on botanical products has grown significantly as described by (Oluwafemi et al., 2020), cattle, goat, sheep, horses and pigs represents about 70% of the animals treated with herbal remedies followed by poultry (9.1%), dogs (5.3%) and rabbits (4.3%). Medicinal plants are mostly used especially by rural and small-scale farmers because they are inexpensive, readily available and effective. Recently, researchers have suggested the use plant extracts and essential oils as alternative growth promoters in poultry feed because they have antimicrobial, anti-parasitic, antioxidant and anti-helminthic properties due to the presence of phytochemical or bioactive chemicals in plants (Oluwafemi et al., 2020).

Fusarium appears to be phytopathogenic, It alters the early stages of development of the ryegrass seedlings tested (Saghrouchni et al., 2021). This genus is capable of invading other host plants with intense virulence, including other grasses and cereals. Treating seedlings with carvacrol has effectively protected them against Fusarium wilt disease. In addition to the anti-Fusarium action, carvacrol is thought to exert a growth-promoting effect on the seedlings. When left untreated, the fungus contained in the seeds becomes fully established. Thus, damaged seeds may harbour masses of conidia, when the seeds were able to germinate, diseased seedlings emerge (Saghrouchni et al., 2021). To control Fusarium, seeds treatment has been recommended (Mironenko, 1960). The alterations observed in seedlings from inoculated seeds show that these species can be troublesome in the early stages of seedling development. Therefore, it is important to monitor their development especially when using susceptible varieties.

## Conclusion

Carvacrol and other plant products may be utilized in controlling diseases like Fusarium wilt, as well as improving plant yield. Further, to understand how carvacrol act as a growth-promotor and biostimulator for enhancing coleoptile growth and rhizogenesis, future studies are recommended. Carvacrol may interact with phytohormones such as auxins, cytokinins, gibberellins, etc. It remains a molecule of plant origin!

## Data availability statement

The raw data supporting the conclusions of this article will be made available by the authors, without undue reservation.

## Author contributions

HS, Writing—original draft preparation; HS, IL, and TA, formal analysis; HS, MB, AB, IM, AA, AH, IV, and HN, writing—review and editing. All authors contributed to the article and approved the submitted version.
